# Prevalence of alcohol use during pregnancy, Brazil, 2011-2012

**DOI:** 10.1590/0102-311XEN232422

**Published:** 2023-08-07

**Authors:** Vanderlea Poeys Cabral, Claudia Leite de Moraes, Francisco I. Bastos, Angela Maria Mendes Abreu, Rosa Maria Soares Madeira Domingues

**Affiliations:** 1 Instituto Nacional de Saúde da Mulher, da Criança e do Adolescente Fernandes Figueira, Fundação Oswaldo Cruz, Rio de Janeiro, Brasil.; 2 Instituto de Medicina Social, Universidade do Estado do Rio de Janeiro, Rio de Janeiro, Brasil.; 3 Instituto de Comunicação e Informação Científica e Tecnológica em Saúde, Fundação Oswaldo Cruz, Rio de Janeiro, Brasil.; 4 Escola de Enfermagem Anna Nery, Universidade Federal do Rio de Janeiro, Rio de Janeiro, Brasil.; 5 Instituto Nacional de Infectologia Evandro Chagas, Fundação Oswaldo Cruz, Rio de Janeiro, Brasil.

**Keywords:** Pregnancy, Alcoholic Beverages, Surveys and Questionnaires, Prevalence, Gravidez, Bebidas Alcoólicas, Inquéritos e Questionários, Prevalência, Embarazo, Bebidas Alcohólicas, Encuestas y Cuestionarios, Prevalencia

## Abstract

This is a national cross-sectional, hospital-based study, which interviewed 23,894 postpartum women in 2011-2012 aiming to estimate the prevalence of alcohol use during pregnancy and identifying more vulnerable groups. Alcohol use during pregnancy was identified using the TWEAK scale, and women with a score of ≥ 2 were classified as having a “presumable diagnosis of inadequate alcohol use”. The national prevalence of alcohol use and the prevalence in subgroups were calculated according to maternal characteristics, with respective 95% confidence intervals (95%CI). Coexistence of smoking, inadequate prenatal consultations, and alcohol use during pregnancy were graphically identified. The prevalence of alcohol use was 14% (95%CI: 13.3-14.7), with 10% (95%CI: 9.3-10.6) of women presenting presumable diagnosis of inadequate alcohol us during pregnancy. Higher prevalence of alcohol use and presumable diagnosis of inadequate alcohol us was observed in black women, aged 12-19 years, with lower educational level, from a lower economic class, without a partner, without paid work, with more than three previous births, who did not want to get pregnant, with inadequate prenatal care, with previous delivery in public services, and who reported smoking during pregnancy. Among the interviewees, 1.2% presented all three risk factors for negative perinatal outcomes at the same time: smoking, alcohol use, and inadequate prenatal care. The results showed a high prevalence of alcohol use during pregnancy and presumable diagnosis of inadequate alcohol us, especially among women with worse social conditions. These data are relevant for the formulation of public policies to prevent alcohol use and provide support services to help this population stop alcohol use during pregnancy.

## Resumo

Estudo transversal, de base hospitalar, nacional, com entrevista de 23.894 puérperas, em 2011-2012, com os objetivos de estimar a prevalência de consumo de álcool na gestação e identificar grupos mais vulneráveis. O uso de álcool na gestação foi identificado por meio da escala TWEAK, sendo classificadas como “diagnóstico presumível de uso inadequado de álcool” mulheres com pontuação ≥ 2. Calculou-se a prevalência nacional de uso de álcool e em subgrupos de acordo com características maternas, com respectivos intervalos de 95% de confiança (IC95%). Foram encontradas, de forma gráfica, coexistência de tabagismo, inadequação de consultas pré-natais e ingestão de bebidas alcoólicas na gestação. A prevalência de uso de álcool foi de 14% (IC95%: 13,3-14,7), com 10% (IC95%: 9,3-10,6) das mulheres apresentando diagnóstico presumível de uso inadequado de álcool na gestação. Maiores prevalências de uso de álcool e de diagnóstico presumível de uso inadequado foram observadas em mulheres pretas, com 12-19 anos de idade, com menor índice de escolaridade, de classe econômica mais baixa, sem companheiro, sem trabalho remunerado, com mais de três partos anteriores, que não queriam engravidar, com assistência pré-natal inadequada, com parto em serviços públicos e que referiram tabagismo na gestação. Estima-se que 1,2% das mulheres entrevistadas apresentavam concomitância dos três fatores de risco para desfechos perinatais negativos: fumo, álcool e assistência pré-natal inadequada. Os resultados demonstraram alta prevalência de uso de álcool na gestação e de diagnóstico presumível de uso inadequado, principalmente por mulheres em situação de vulnerabilidade social. São relevantes a elaboração de políticas públicas que contemplem ações de prevenção do uso de bebidas alcoólicas e a prestação de serviços de apoio para cessação do uso de álcool na gravidez.

## Resumen

Estudio transversal, de base hospitalaria, nacional, con entrevistas a 23.894 puérperas, en 2011-2012, con el objetivo de estimar la prevalencia de consumo de alcohol durante el embarazo e identificar grupos más vulnerables. El consumo de alcohol durante el embarazo se identificó mediante la escala TWEAK, y las mujeres con puntuación ≥ 2 fueron clasificadas como “diagnóstico presumible de uso inadecuado de alcohol¨. Se calculó la prevalencia nacional de consumo de alcohol y subgrupos según características maternas, con sus respectivos intervalos de 95% de confianza (IC95%). Se identificó gráficamente la coexistencia de tabaquismo, consultas prenatales inadecuadas y consumo de alcohol durante el embarazo. La prevalencia de consumo de alcohol fue del 14% (IC95%: 13,3-14,7), siendo el 10% (IC95%: 9,3-10,6) de mujeres con diagnóstico presumible de uso inadecuado durante el embarazo. Se observaron mayores prevalencias de consumo de alcohol y diagnóstico presumible de uso inadecuado en mujeres de color/raza negra, de 12-19 años, con menos años de escolaridad, de clase económica más baja, sin pareja, sin trabajo remunerado, con más de tres partos previos, que no querían quedar embarazadas, con control prenatal inadecuado, con parto en el sistema público de salud y que relataron fumar durante el embarazo. Se estima que el 1,2% de las mujeres entrevistadas presentaron la concomitancia de tres factores de riesgo en resultados perinatales negativos: tabaquismo, alcohol y atención prenatal inadecuada. Los resultados demuestran una alta prevalencia de consumo de alcohol durante el embarazo y de diagnóstico presumible de uso inadecuado, especialmente en mujeres con las peores condiciones sociales, siendo relevante para la elaboración de políticas públicas que incluyan acciones de prevención del consumo de alcohol y servicios de apoyo para el cese del consumo de alcohol en el embarazo.

## Introduction

Alcohol use during pregnancy is an important public health problem, and it is associated with negative maternal and fetal outcomes ^1^. The effects on maternal health include bleeding during pregnancy, miscarriage, premature birth, and placental abruption ^2^ ^,^ ^3^ ^,^ ^4^. For the fetus, excessive exposure to prenatal alcohol is associated with teratogenic effects and the development of fetal alcohol syndrome (FAS) ^5^. In Brazil, estimates suggest that around 1,500 to 6,000 children are born with FAS every year ^6^.

Alcohol consumption is a modifiable risk behavior during pregnancy and, although the scientific evidence on the effects of light and moderate consumption is not conclusive ^7^ ^,^ ^8^, most guidelines recommend that pregnant women, or those intending to become pregnant, abstain from drinking any amount of alcohol, as it is known to be a teratogenic exposure, and because there is still no consensus on the level of consumption that could be considered safe ^1^ ^,^ ^9^ ^,^ ^10^ ^,^ ^11^.

Despite these recommendations, recent estimates suggest that 9.8% (95% confidence interval - 95%CI: 8.9-11.1) of the global population of pregnant women drink alcohol in the prenatal period ^5^. In Europe, the region with the highest estimate of alcohol use during pregnancy, the point prevalence is 25.2% (95%CI: 21.6-29.6) ^5^. In Central America, South America, and the Caribbean, insufficient data are found about alcohol use by pregnant women. However, estimates indicate excessive exposure, with rates above the average found in other countries in the world, as observed in Grenada, in the Caribbean (23.3%) ^12^. In Brazil, national-level estimates of alcohol use during pregnancy are not available, with only local studies estimating the prevalence of alcohol use during pregnancy, which ranged from 1.8% in a maternity hospital in Bahia State to 40.6% in three maternity hospitals analyzed together in the city of Rio de Janeiro ^5^.

A complex group of sociodemographic characteristics, clinical and obstetric history, prenatal care, and behavioral data can be associated with alcohol use in pregnancy, such as skin color (a common proxy, although not very accurate for ethnicity) ^13^ ^,^ ^14^ ^,^ ^15^, economic class ^16^, paid work ^13^, history of chronic disease ^17^, parity ^14^ ^,^ ^15^, intended pregnancy ^18^, number of prenatal consultations ^16^, and intimate partner violence ^19^ ^,^ ^20^. Some of them have well-established associations, such as smoking ^21^, while others, like marital status and educational level, show divergence ^14^ ^,^ ^15^ ^,^ ^16^ ^,^ ^17^.

Based on these individual variables showing unequal access to policies and services - making them vulnerable subjects ^22^ - we attempted to estimate the prevalence of alcohol use during pregnancy according to maternal characteristics in order to support the development of prevention and control strategies. This study aimed to estimate the national prevalence of alcohol use during pregnancy and identify groups that are more exposed to this problem, according to maternal sociodemographic, obstetric, and behavioral characteristics.

## Method

### Study design

This is a national, hospital-based study on pregnancy, delivery, and birth care conducted in 2011-2012 and titled *Birth in Brazil* survey. The sample of the *Birth in Brazil* survey was calculated considering the proportion of cesarean sections in Brazil in 2007 of 46.6% and a confidence level of 5% to detect differences of 14% between public and mixed hospitals and private hospitals. A 1.3 design effect ^23^ was used, resulting in a planned sample of 23,940 postpartum women from 266 hospitals in all states of the country. 

The sampling process comprised three stages of selection. In the first stage, hospitals with 500 or more annual deliveries were selected, stratified according to the country’s macro regions, location (capital or non-capital), and type of hospital (public, private, or mixed), with a probability of selection proportional to the number of deliveries in each of the strata in 2007. In the second stage, the number of days needed to interview 90 postpartum women in every hospital (minimum of seven days) was defined using an inverse sampling method. In the third and final stage, eligible postpartum women were selected. More information about the sample design is detailed in Vasconcellos et al. ^24^.

### Participants

Postpartum women with hospital live births of any weight and gestational age or stillbirths with birth weight ≥ 500g and/or gestational age ≥ 22 weeks were considered eligible for the study. Women who gave birth at home, on a public street, or at another health institution that was not part of the sample; women with serious psychiatric illness or foreigners who did not understand Portuguese; and women with hearing impairment were considered ineligible for the study.

### Data collection

Participants were interviewed during their hospital stay in the immediate postpartum period by a team of trained interviewers. Data from the hospital records of every woman and newborn were extracted after hospital discharge. Prenatal cards were photographed, with subsequent data extraction.

### Study variables

To assess alcohol use during pregnancy, the Brazilian version of the Tolerance, Worry, Eye-opener, Amnesia/black-out, and K/Cut Down (TWEAK) ^19^ instrument was used. This instrument consists of five questions: T - *tolerance =* “how many drinks can you hold?”; W - *worried* = “have close friends or relatives worried or complained about your drinking in the past year?”; E - *eye-opener* = “do you sometimes take a drink in the morning when you first get up?”; A - *amnesia - stands for blackout*s = “has a friend or family member ever told you about things you said or did while you were drinking that you could not remember?”; K - *cut down* = “do you sometimes feel the need to cut down on your drinking?” ^19^. A positive response to the “tolerance” question (three or more drinks without falling asleep or passing out) and a positive response to the “worry” question receive two points each, while a positive response to each of the last three questions receives one point each, with scores ranging from zero to seven points. Women who did not report alcohol use during pregnancy were classified as “did not use alcohol during pregnancy”, women with a TWEAK score of less than two points were classified as “no inappropriate alcohol use”, and women with two or more points in the total score were classified as “presumable diagnosis of inappropriate alcohol use”, using the same nomenclature adopted by the authors who validated the scale in Brazil ^13^. This cutoff point, which is suggested by the original scale validation study, has 70%-90% sensitivity and 65%-90% specificity ^25^ ^,^ ^26^.

The following maternal characteristics were analyzed: (a) demographic and socioeconomic: geographic region of residence of the participant (North, Northeast, Central-West, South, and Southeast); maternal age (12-19, 20-34, and 35 older), self-reported race/skin color (white, black, mixed-race, “yellow” [following IBGE criteria], indigenous); educational level in years (up to 7, 8-10, 11 or more years); marital status (without a partner, with a partner); economic class (D+E, C, A+B, where A/B are the highest classes) ^27^, paid work (yes or no); type of childbirth procedures funding (public or private); (b) obstetric history: parity (0, 1-2, 3 or more); (c) current pregnancy data: intended pregnancy (I wanted to get pregnant now; I wanted to get pregnant, but not now; I didn’t want to get pregnant); start of prenatal care (first, second, third trimester of pregnancy), adequacy of the number of prenatal consultations for gestational age at delivery (inadequate, partially adequate, adequate, more than adequate); (d) behavioral: smoking during pregnancy (yes or no).

Data about age, education, economic class, marital status, work, childbirth procedures funding, intended pregnancy, smoking, and alcohol use were obtained in the interviews with postpartum women. Data about the obstetric history and prenatal care were obtained mainly from the prenatal card, and data from hospital records and structured interviews with postpartum women were also used when the card was not available.

The adequacy of the number of prenatal consultations was evaluated considering the gestational age at delivery and the schedule of consultations recommended by the Brazilian Prenatal Humanization Program ^28^ in effect at the time of the study: one consultation in the first trimester of pregnancy, two in the second trimester of pregnancy, and three in the third trimester of pregnancy, totaling at least six consultations for a full-term pregnancy.

### Data analysis

The prevalence of the two categories of alcohol use and their respective 95%CI were estimated in the sample as a whole and in the categories of maternal variables. Statistical analyses were performed using R (version 4.1.2, http://www.r-project.org) and its Survey library, including weighting, calibration, and design effect in all stages of the statistical analysis ^28^.

A Venn diagram ^29^ was used to assess the coexistence of maternal characteristics that may be associated with a higher risk of negative perinatal outcomes. In addition to alcohol use during pregnancy, smoking during pregnancy and the adequacy of the number of consultations were analyzed. The variable “smoking during pregnancy” was selected due to its known effect on perinatal outcomes, such as low weight, prematurity, and malformations ^21^. The variable “adequacy of the number of prenatal consultations” was selected because it is an important component of such management and care, associated with better perinatal outcomes when in adequate number ^30^. In addition, a higher number of prenatal service consultations can provide greater opportunity for actions to reduce smoking and alcohol use during pregnancy, which are modifiable risk factors with good response to brief interventions ^31^.

### Ethical aspects

This study uses data from the *Birth in Brazil* survey approved by the Research Ethics Committee of the Sergio Arouca National School of Public Health, Oswaldo Cruz Foundation (ENSP/Fiocruz; CAAE 0096.0.031.000-10/June 10, 2010). All precautions were adopted to ensure the data secrecy and confidentiality. Before conducting every interview, participants signed an informed consent form.

## Results

Of all 23,894 postpartum women interviewed in the *Birth in Brazil* survey, most lived in the Southeast (42.5%) and Northeast (28.9%) regions of Brazil. The mean age was 27 years, with 70.4% of the women in the 20-34 age group. Most interviewees reported mixed race/skin color (56.1%), with a small proportion of women reporting “yellow” (1.1%) and indigenous (0.4%) ethnicity. Almost half (47.8%) of the participants had 11 years or more of education, 52% belonged to the “C” class, 81.4% lived with a partner, 59.7% did not have paid work, and 80.1% gave birth in the public health system. Regarding the current pregnancy, 46.9% were primiparous, 44.3% wanted to get pregnant, 60.6% started prenatal care in the first trimester of pregnancy, and more than half of the total interviewees had an adequate number of prenatal care consultations, considering the number of consultations and their gestational age at delivery. Of the total number of participants, 9.6% reported smoking during pregnancy (Table 1).

**Table 1:** Demographic, socioeconomic, obstetric, and behavioral characteristics of postpartum women. Brazil, 2011-2012 (N = 23,894).

Maternal characteristics	%	95%CI
Region of residence		
North	9.6	9.6-10.3
Northeast	28.9	27.3-37.5
Southeast	42.5	40.3-44.7
South	12.5	11.3-13.4
Central-West	6.5	5.7-7.5
Age (years)		
12-19	19.1	18.7-19.6
20-34	70.4	69.9-70.8
35 older	10.5	10.2-10.8
Skin color		
White	33.8	32.9-34.7
Mixed-race	56.1	55.2-57.0
Black	8.6	8.1-9.1
Yellow	1.1	0.9-1.2
Indigenous	0.4	0.3-0.5
Educational level (years of education)		
11 or more	47.8	46.9-48.7
8-10	25.6	25.1-26.1
Up to 7	26.6	25.9-27.3
Economic class		
A+B	24.3	23.5-25.1
C	52.0	51.2-52.8
D+E	23.7	23.0-24.4
Marital status		
With a partner	81.4	81.0-81.9
Without a partner	18.6	18.1-19.0
Paid work		
Yes	40.3	39.6-40.9
No	59.7	59.1-60.4
Delivery service		
Private	19.9	19.2-20.7
Public	80.1	79.3-80.8
Parity		
0	46.9	46.4-47.4
1-2	42.7	42.3-43.2
3 or more	10.4	10.0-10.7
Intended pregnancy		
I wanted to get pregnant now	44.3	43.7-44.8
I wanted to get pregnant, but not now	25.4	24.9-25.8
I didn’ t want to get pregnant	29.7	29.1-30.2
Beginning of prenatal care (gestational trimester)		
1st	60.6	59.7-61.9
2nd	35.8	34.5-37.1
3rd	3.7	3.3-4.1
Adequacy of the number of prenatal consultations		
More than adequate	19.3	18.7-19.9
Adequate	33.9	33.3-34.6
Partially adequate	26.4	26.0-26.9
Inadequate	20.3	19.8-20.9
Smoking in the current pregnancy		
Yes	9.6	9.3-9.9
No	90.4	90.1-90.7

95%CI: 95% confidence interval.

Alcohol use during pregnancy was estimated at 14% (95%CI: 13.3-14.7), with 10% (95%CI: 9.4-10.6) of women presenting presumable diagnosis of inappropriate alcohol use during pregnancy (Table 2). A lower prevalence of total alcohol use was observed in pregnant women living in the North and Northeast regions, while a lower presumable diagnosis of inappropriate alcohol use of alcohol was observed only in the North Region. Higher alcohol consumption and presumable diagnosis of inappropriate alcohol use were observed in women under 35 years old, black, with up to ten years of education, from economic classes “C” and “D+E”, without a partner, without paid work, patients of the public health system, with three children or more, who did not want to get pregnant, with prenatal care beginning in the second or third trimester of pregnancy, with an inadequate number of prenatal consultations, and who smoked during pregnancy; smokers presented a prevalence that was over three times the prevalence among non-smokers.

**Table 2:** Total use and presumed diagnosis of inappropriate alcohol use during pregnancy according to demographic and socioeconomic characteristics, obstetric history and data from the current pregnancy. Brazil, 2011-2012 (N = 23,894).

Maternal characteristic	Total alcohol use		Presumable diagnosis of inadequate alcohol use	
	%	95%CI	%	95%CI
Total	14.0	12.7-15.4	10.0	9.4-10.6
Region of residence *				
North	8.6	6.9-10.2	7.5	5.9-9.1
Northeast	12.0	10.8-13.1	9.7	8.7-10.7
Southeast	14.6	13.3-15.8	10.4	9.4-11.4
South	19.6	17.8-21.4	10.7	9.7-11.7
Central-West	16.4	14.0-18.7	11.6	9.9-13.3
Age (years) *				
12-19	14.5	13.5-15.5	11.3	10.4-12.2
20-34	14.2	13.4-14.9	10.2	9.6-10.8
35 older	11.9	10.9-12.8	6.5	5.8-7.2
Skin color *				
White	12.2	11.4-13.0	7.6	6.9-8.3
Mixed-race	13.9	13.2-14.7	10.4	9.8-11.0
Black	21.0	19.2-22.8	16.5	14.9-18.1
Yellow	15.3	12.8-17.8	13.6	11.2-16.0
Indigenous	11.8	7.5-16.1	11.0	6.8-15.2
Educational level (years of education) *				
11 or more	10.8	10.2-11.3	6.8	6.3-7.3
8-10	16.8	15.7-17.9	12.6	11.7-13.5
Up to 7	17.2	16.2-18.2	13.4	12.5-14.3
Economic class *				
A+B	12.2	11.3-13.0	7.1	6.5-7.7
C	14.6	13.8-15.4	10.8	10.1-11.5
D+E	14.7	13.7-15.7	11.5	10.7-12.3
Marital status *				
With a partner	12.8	12.1-13.5	8.8	8.3-9.3
Without a partner	19.2	18.1-20.3	20.3	14.2-16.4
Paid work *				
Yes	13.4	12.6-14.1	8.6	7.9-9.3
No	14.4	13.6-15.2	11.0	10.3-11.7
Delivery servisse *				
Private	8.1	7.3-8.8	4.5	3.9-5.1
Public	15.5	14.7-16.3	11.4	10.7-12.1
Parity *				
0	12.8	12.0-13.5	9.3	8.7-9.9
1-2	14.1	13.3-14.8	9.7	9.1-10.3
3 or more	19.1	17.7-20.6	14.9	13.6-16.2
Intended pregnancy *				
I wanted to get pregnant now	11.5	10.8-12.1	7.7	7.2-8.2
I wanted to get pregnant, but not now	11.5	8.3-14.7	10.4	9.5-11.3
I didn’ t want to get pregnant	17.7	16.7-18.6	13.4	12.5-14.3
Beginning of prenatal care (gestational trimester)				
1st	12.4	11.1-13.8	8.5	7.4-9.7
2nd	15.7	14.0-17.5	11.6	10.2-13.0
3rd	19.0	15.5-23.1	14.8	11.7-18.6
Adequacy of the number of prenatal consultations *				
More than adequate	11.0	9.5-12.8	7.0	6.2-7.8
Adequate	12.2	10.7-13.9	8.6	8.0-9.2
Partially adequate	15.1	13.3-17.0	10.8	10.0-11.6
Inadequate	18.2	15.8-18.8	14.4	13.3-15.5
Smoking in the current pregnancy *				
Yes	37.1	33.9-40.4	30.6	29.0-32.2
No	11.6	10.4-12.9	7.9	7.4-8.4

95%CI: 95% confidence interval.

* p-value of the chi-square test, significant at 5%.

A gradient of prevalence was observed according to age, educational level, economic class, parity, intended pregnancy, beginning of prenatal care, and adequacy of the number of prenatal consultations, both for alcohol use and presumable diagnosis of inappropriate alcohol use during pregnancy, with a higher prevalence in younger women, with lower educational level, from a lower economic class, who did not want to get pregnant, with a later start of prenatal care and inadequate number of consultations.

**Figure 1:**
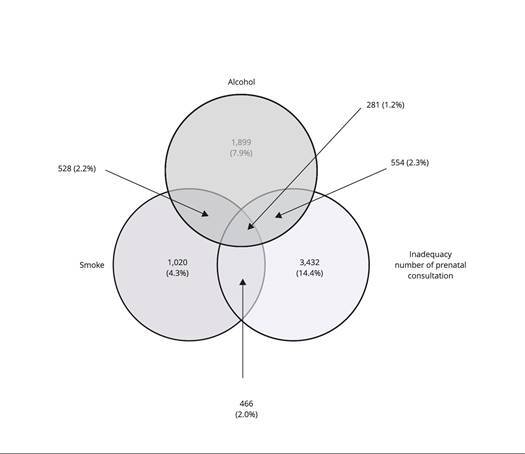
Prevalence of smoking, alcohol use, and inadequate prenatal consultations. Brazil, 2011-2012 (N = 23,894).

The Venn diagram graphically represented the coexistence of all maternal factors analyzed in this study. Of the total study population, 7.9% reported only alcohol consumption; 2.2% smoking and alcohol use; 2.3% alcohol use and inadequate number of consultations; and 1.2% presented the three factors (Figure 1).

## Discussion

The results of this study show that 14% of pregnant women used different amounts of alcohol during pregnancy, with 10% classified as having presumable diagnosis of inappropriate alcohol use. A higher prevalence of total alcohol use during pregnancy was observed in the South Region, and a lower prevalence in the North and Northeast regions, indicating regional differences in alcohol consumption, in a country with significant regional, demographic, and social diversity ^32^. Our study shows an even higher frequency of smoking in women who used alcohol during pregnancy, exposing pregnant women and fetuses to the harmful effects of alcohol and tobacco. Higher prevalence of total alcohol use and presumable diagnosis of inappropriate alcohol use was also observed in women presenting greater social vulnerability, including women with lower educational level, from lower economic classes, non-whites, without a partner during pregnancy, without paid work, with a higher number of previous deliveries, with unintended pregnancies, treated in the public health system, with a late start of prenatal care and an inadequate number of consultations.

A comparison of our results with those of other studies must be performed with caution, as a wide variety of terms are used in the literature to refer to alcohol use during pregnancy ^33^, making it difficult to identify and standardize scientific studies on the subject. In addition to varied terms, different scales have been used to measure alcohol consumption during pregnancy, since there is no universal recommendation regarding the scale to be used. The use of different scales also makes it difficult to compare the estimates and is one of the possible explanations for different prevalence rates observed in studies using distinct scales.

This estimates obtained in our study for total alcohol consumption are higher than global estimates, but lower than those observed in European countries, which show the highest prevalence in the world ^5^. In Latin America and the Caribbean, a meta-analysis of studies conducted in 1984-2014 to assess any alcohol consumption during pregnancy identified 24 studies, obtaining a combined prevalence of alcohol consumption during pregnancy in two countries: Brazil (15.2%, 95% CI: 10.4-20.8) and Mexico (1.2%; 95%CI: 0.0-2.7). For another 31 countries in Latin America and the Caribbean, the prevalence of alcohol consumption during pregnancy ranged from 4.8% (95%CI: 4.2-5.4) in Cuba to 23.3% (95%CI: 20.1-26.5) in Grenada ^12^. Regarding the methods of the studies included in the meta-analysis, 88% used non-probability sampling and only 13% used a validated instrument to investigate alcohol use. Of 19 studies conducted in Brazil, only five used a scale to measure alcohol consumption, and only one study used TWEAK ^13^. An explanation for the high prevalence of alcohol use during pregnancy in some countries in Latin America and the Caribbean is the marketing campaigns advertising alcohol consumption, promoting existing alcohol industries, and the failure to adopt important public policies supported by organizations such as the World Health Organization (WHO) ^34^.

The estimate of 10% presumable diagnosis of inappropriate alcohol use found in our study is close to values reported in the literature, such as in Rio de Janeiro, Brazil, in 2007 (7.3%; 95%CI: 5.1-9.5) ^13^, in Rio Grande do Sul State, Brazil, in 2007/2008 ^35^ (2.1%, 95%CI not reported), and Goiás State, Brazil, in 2014/2015 (17.7%; 95%CI: 14.1-22.0) ^17^. In studies with small samples that used the TWEAK scale, the estimated prevalence of inadequate alcohol use in the world ranged from 13.6% in Italy in 2017 ^36^ to 54% in Canada in 2006 and 2007 ^37^, probably due to the specific characteristics of studied populations. A study with 11,909 pregnant women in Western Ukraine estimated the prevalence of 10.9% of harmful use of alcohol from 2007 to 2012 ^38^, a similar rate to that found in our study. However, the cutoff point used for “tolerance” in the questionnaire was different - six or more drinks without falling asleep or passing out. If the two studies in question had used three drinks or more as the cutoff point, 52.2% of women in Western Ukraine would have presented presumable diagnosis of inappropriate alcohol use during pregnancy.

The prevalence of presumable diagnosis of inappropriate alcohol use during pregnancy was higher in subgroups already identified in the literature: young women ^17^ of lower educational level ^16^, low economic class ^13^ ^,^ ^14^, non-whites ^13^, without a partner ^13^, with unintended pregnancy ^13^ ^,^ ^18^ ^,^ ^39^ and multiparity ^40^, without paid work ^13^, with inadequate prenatal care ^15^, and who reported smoking during pregnancy ^15^ ^,^ ^17^.

Indigenous women had a higher prevalence rate of alcohol use when compared to white women, but no significant difference was observed in relation to mixed-race and “yellow” women, probably due to the small sample size of indigenous people in the study. This finding should be explored in future studies with this specific population, as the excessive use of alcohol has been reported in other studies and institutional documents, especially among indigenous people living outside the village, in urban peripheries ^41^.

A higher prevalence of alcohol consumption in pregnant women living in the South Region and a lower prevalence in pregnant women living in the North Region are consistent with national studies assessing the general population ^42^. In a study conducted by Bastos et al. ^42^, which assessed a population aged over 12 years, the prevalence of alcohol consumption was 22.2% in the North Region and 32.5% in the South Region. In 2019, in the *Brazilian National Health Survey* (PNS), which evaluated the population aged 18 years and older, point prevalence rates for these regions were 20.5% and 35.6%, respectively. These data suggest that a regional pattern of alcohol use in adults that is reproduced during pregnancy.

On the other hand, when analyzing the prevalence of alcohol use according to educational level, our findings do not agree with national studies that assessed adolescents and/or adults outside the gestational period. While in our study the prevalence was higher in women with lower educational level, national studies assessing participants outside the gestational period indicate that higher educational levels are linked with higher use of alcohol ^42^ ^,^ ^43^ ^,^ ^44^. This pattern is observed both in men and in non-pregnant women. Regarding the consumption of four drinks or more on a single occasion (binge drinking), the most recent Brazilian telephone survey ^45^ showed that, among women, alcohol consumption increased with the educational level. In Denmark, a higher occurrence of binge drinking was also observed in women with higher educational level before the diagnosis of pregnancy ^46^.

One hypothesis for the higher consumption of alcohol during pregnancy among women with lower educational level in Brazil is the poor access to information about the harmful effects of alcohol use during pregnancy. Studies in Africa reporting a higher prevalence of alcohol consumption during pregnancy, including binge drinking, in pregnant women with fewer years of education ^47^, or among residents in communities with lower educational level ^48^, found a much higher chance of these women not being aware of the risks of alcohol use during pregnancy ^47^ and considering alcohol use during pregnancy as socially or culturally accepted, than that observed in women with higher educational level ^48^.

The difference observed in alcohol consumption between women of higher and lower educational levels suggests that different strategies are required, with actions for the general population, focused on women of higher educational levels, and prenatal care actions especially for women of lower educational levels.

A higher prevalence of alcohol use was observed in women with inadequate number of prenatal consultations. Insufficient consultations offer fewer opportunities for counseling about the importance of not using alcohol during pregnancy, whether in individual consultations or educational activities related to the subject. Lower use of prenatal services by women who use alcohol may be due to different reasons, including higher social vulnerability, obstacles to visiting health services, and low adherence to health promotion activities, such as prenatal care.

Increased use of alcohol and smoking and the inadequate number of prenatal consultations associated with negative outcomes for the newborn reinforces the importance of further expanding access to prenatal care for the most vulnerable women. These three factors can be an alert for efforts to prevent and monitor this population of pregnant women who present these risk factors. The identification of a higher prevalence of alcohol use in women with late beginning of prenatal care also highlights the need for strategies to promote an early diagnosis of pregnancy, avoiding exposure to alcohol when the woman does not know she is pregnant. Data from the *Birth in Brazil* survey show that 46.6% of women with late beginning of prenatal care mentioned challenges in pregnancy diagnosis as a reason for not having started prenatal care earlier ^30^.

The results of this study must be interpreted considering its limitations and strengths. Study limitations include the fact that estimates cannot be extrapolated to women who gave birth at home, on public roads, in hospitals with less than 500 births a year, and pregnant women whose pregnancy miscarriage, since they were not eligible for the study. Another possible limitation was the method to measure the inappropriate use of alcohol through self-report and retrospectively. Retrospective data collection may underestimate the use of alcohol in early pregnancy, when the woman does not know she is pregnant ^49^. Self-report, on the other hand, may underestimate the use of alcohol during pregnancy due to the embarrassment of women in admitting its use, given the concern about the harmful effects of alcohol during pregnancy for the fetus and newborn ^50^ ^,^ ^51^. However, using the TWEAK questionnaire may have mitigated these limitations, as it is a scale developed for use in obstetrics and gynecology clinics and in primary care ^1^, ensuring high sensitivity and specificity in identifying the inappropriate use of alcohol during pregnancy in different ethnic groups ^26^ ^,^ ^51^. The absence of information about gestational age in the diagnosis of pregnancy, social support during pregnancy, and guidance received during pregnancy regarding the use of alcohol during pregnancy and its harmful effects also limit the understanding of the importance of these factors related to alcohol consumption during pregnancy. A study conducted in Australia showed a low proportion of counseling received by pregnant women, despite existing care guidelines ^52^. In Brazil, two studies assessed prenatal counseling ^53^ ^,^ ^54^ and reported that women consider it is important to be informed about the dangers of consuming alcohol during pregnancy, but they received little information about that, sometimes incorrectly, and not always easy to understand. A relationship full of conflict with the partner has also been reported as one of the factors associated with higher alcohol consumption during pregnancy ^55^ ^,^ ^56^, as well as social reasons for drinking during pregnancy ^54^ ^,^ ^57^. Finally, contextual factors have also been evaluated, such as living in an environment that does not encourage physical activity ^58^. None of these factors was available in our study and should be explored in future investigations.

In terms of study strengths, this is the first study that estimated the prevalence of alcohol use and presumable diagnostic of inapproriate alcohol use during pregnancy that used a comprehensive and representative sample of the country and all its regions. Although data refer to 2011-2012 and may not reflect current prevalence, they present a national scenario of alcohol use during pregnancy, and regional and social inequalities that can support the formulation of health policies, which must be updated with new studies. Specific policies on alcohol consumption during pregnancy have not been implemented since then, and it is unlikely that changes have been made in service routines. Regarding the inequalities observed in alcohol consumption, data from the PNS conducted in 2013 and 2019 ^43^ ^,^ ^44^ estimate an increase in alcohol consumption of once or more per week in women, residents in the Southeast region, and people presenting higher educational level. However, these changes cannot be extrapolated to the population of pregnant women and the assessment of inequalities in consumption among pregnant women depends on further specific studies.

## Conclusion

Around 14% of Brazilian pregnant women reported alcohol use during pregnancy and 10% presented presumable diagnosis of inadequate alcohol use during pregnancy, with higher prevalence of alcohol use among women in higher social vulnerability and among smokers. These results may be relevant for the development of public policies and care guidelines that include actions to improve prenatal care, prevent alcohol use, and offer support services to stop alcohol use during pregnancy. Screening and counseling on alcohol consumption during pregnancy - actions recommended by the WHO ^1^ - should be implemented in all prenatal services, as well as educational actions addressing the risk of alcohol use, with a focus on all pregnant women, especially those who are more vulnerable.
